# A canine model of Cohen syndrome: Trapped Neutrophil Syndrome

**DOI:** 10.1186/1471-2164-12-258

**Published:** 2011-05-23

**Authors:** Jeremy R Shearman, Alan N Wilton

**Affiliations:** 1 School of Biotechnology and Biomolecular Sciences, University of New South Wales, Sydney, NSW 2052, Australia; 2 National Center for Genetic Engineering and Biotechnology, 113 Phahonyothin Rd., Klong 1, Klong Luang, Pathumthani 12120, Thailand; 3 Clive and Vera Ramaciotti Centre for Gene Function Analysis, University of New South Wales, Sydney, NSW 2052, Australia

**Keywords:** Trapped Neutrophil Syndrome, Cohen Syndrome, neutropenia, vesicle transport, expression, vesicle protein sorting 13 B, VPS13B, dog

## Abstract

**Background:**

Trapped Neutrophil Syndrome (TNS) is a common autosomal recessive neutropenia in Border collie dogs.

**Results:**

We used a candidate gene approach and linkage analysis to show that the causative gene for TNS is *VPS13B*. We chose *VPS13B *as a candidate because of similarities in clinical signs between TNS and Cohen syndrome, in human, such as neutropenia and a typical facial dysmorphism. Linkage analysis using microsatellites close to *VPS13B *showed positive linkage of the region to TNS. We sequenced each of the 63 exons of *VPS13B *in affected and control dogs and found that the causative mutation in Border collies is a 4 bp deletion in exon 19 of the largest transcript that results in premature truncation of the protein. Cohen syndrome patients present with mental retardation in 99% of cases, but learning disabilities featured in less than half of TNS affected dogs. It has been implied that loss of the alternate transcript of *VPS13B *in the human brain utilising an alternate exon, 28, may cause mental retardation. Mice cannot be used to test this hypothesis as they do not express the alternate exon. We show that dogs do express alternate transcripts in the brain utilising an alternate exon homologous to human exon 28.

**Conclusion:**

Dogs can be used as a model organism to explore the function of the alternately spliced transcript of VPS13B in the brain. TNS in Border collies is the first animal model for Cohen syndrome and can be used to study the disease aetiology.

## Background

Dogs consist of over 400 genetically isolated breeds with considerable morphological and behavioural diversity. Dog breeds have undergone two major bottlenecks, the first when they were domesticated from the wolf ~15,000 years ago [1,2], and the second in the last few hundred years during development of the modern breeds from a low number of individuals selected for certain physical or behavioural traits [1,3]. Such heavy artificial selection results in limited genetic variation within each breed and many inherited diseases. Inherited diseases in dogs are predominantly recessive, often resulting from strong inbreeding and usually showing allelic homogeneity within a breed or group of related breeds and allelic heterogeneity between less related breeds [4].

An autosomal recessive neutropenia, known as Trapped Neutrophil Syndrome (TNS), has been identified in the Border collie breed. The disease was originally described in the Australian and New Zealand population of Border collies and is characterised by a deficiency of segmented neutrophils in the blood and hyperplasia of myeloid cells in the bone marrow [5]. Severely affected pups show abnormal craniofacial development with a narrowed elongated skull shape described by breeders as ferret-like. Affected pups are often smaller than their litter mates and suffer from chronic infections and failure to thrive resulting from a compromised immune system. Some show early infections from six weeks of age. For others the first sign of TNS is a bad reaction to immunisation at 12 weeks, while in a few cases clinical signs are very mild and not recognised until two or more years of age [5-7].

All known TNS affected dogs at the start of this research could be traced back to a single common ancestor [7]. This suggested that the disease was the result of a single mutational event that had been spread through the population by the champion sire effect. Champion male show dogs are often used heavily for breeding to pass on show winning traits. Any detrimental mutations that a champion male may be carrying can rapidly spread through a population. When coupled with inbreeding, recessive diseases can manifest within a few generations after the champion is used. All affected dogs are then homozygous and identical-by-descent for the genomic region surrounding the mutation. We considered allelic heterogeneity unlikely for TNS given the recent common ancestor.

A promising approach to identify disease genes is the candidate gene approach coupled with linkage analysis using microsatellites from the candidate gene region. This approach can confirm whether the region is the correct one even if the mutation is non-coding. We chose candidate genes in this study based on their role in pathways known to be associated with neutrophil maturation or known to cause neutropenia in humans.

We investigated 10 genes as candidates for TNS, four of these are known to cause a disease featuring neutropenia and six are linked to neutrophil function. We have previously excluded six of the genes from this list: chemokine (C-X-C motif) receptor 4 (*CXCR4*), [6]; neutrophil elastase (*ELANE*); adaptor-related protein complex 3, beta 1 subunit (*AP3B1*); and adaptor-related protein complex subunits *AP3D1*, *AP3S1 *and *AP3M1 *[7].

One of the other four candidates (Table 1) was the ligand of *CXCR4*, chemokine (C-X-C motif) ligand 12 (*CXCL12*), which may result in a similar phenotype to disruption of *CXCR4*. Another candidate was the anti-apoptotic factor, BCL2-like 1 (*BCL2L1*), which has been shown to be down-regulated in cases of neutropenia [8]. We also investigated interleukin 8 receptor B (*CXCR2*) as it is able to attract and activate neutrophils to sites of inflammation from the blood [9]. We considered Cohen syndrome (MIM:216550) [10,11] as a model for TNS because of presence of the clinical signs of neutropenia with hyperplasia of the bone marrow enriched for the myeloid cell lines [12] and a typical craniofacial dysmorphism. Cohen syndrome is caused by mutations in Vesicle Protein Sorting 13B (*VPS13B*). We used linkage analysis followed by sequencing to investigate whether a mutation in one of these genes is responsible for TNS in Border collies.

**Table 1:** LOD score for linkage analysis using microsatellites in the region of three candidate genes for TNS not previously reported.

Candidate	Name	LOD	location
*BCL2L1*	*BCL-XL*	-6.05	Chr 24, 24.1 Mb

*CXCL12*	*stromal cell derived factor 1*	-5.90	Chr28, 58.9 Mb

*CXCR2*	*Interleukin 8 receptor*	-3.34	Chr37, 27.9 Mb

## Results

We examined the four candidate genes (Table 1) for linkage to TNS using between one and five microsatellites for each candidate. We eliminated three from being linked to TNS based on maximum logarithm of odds (LOD) scores less than minus-two from multipoint linkage analysis in an eight generation pedigree with seven to 12 affecteds depending on the analysis (Table 1). We developed genotyping assays for four microsatellites from the *VPS13B *region and named them C13.0390, C13.0423, C13.0449 and C13.0478 based on chromosome number and location in 10's of kb (Additional file 1, table S1). These microsatellites were highly polymorphic with between eight and 14 alleles in a sample of 72 Border collies (Additional file 1, table S2). We observed 23 haplotypes involving alleles at these loci and a large amount of linkage disequilibrium between alleles (Additional file 1, table S3). Multipoint linkage analysis for our TNS pedigree, with multiple inbreeding loops [7], gave significant evidence of linkage with a maximum LOD score of 8.86 at marker C13.0390 (Additional file 1, figure S1).

We observed strong linkage disequilibrium between TNS and the microsatellites at *VPS13B*, with the majority of affected dogs homozygous for a single haplotype (Additional file 1, figure S2). Variations in the disease haplotype occurred in three dogs, each at 1 locus only and likely represent new mutations in the microsatellite rather than recombinants.

**Figure 1:**
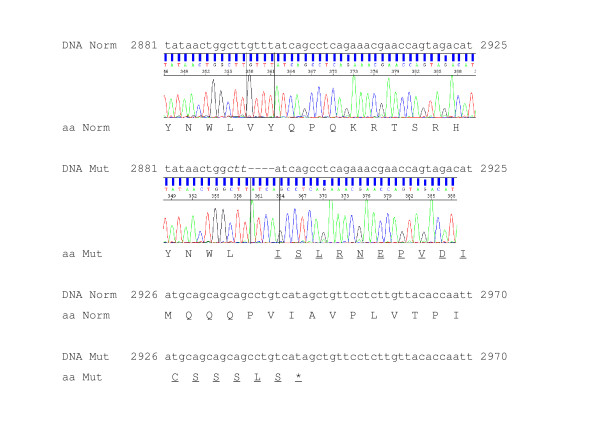
**Partial DNA sequence, sequence electropherogram and amino acid sequence for *VPS13B *region containing TNS mutation for normal (Norm) and mutant (Mut) genomic sequence (above) with translation (below)**. Underlined aa represent expected aa after the frameshift due to deletion, stop codon represented by *. Number on DNA sequence represents expected location on cDNA of isoform variant 1

We sequenced each of the 63 exons with at least 100 bp of flanking intron in two controls, two obligate carriers and two affecteds. We found 13 coding sites that were either polymorphic in these dogs or different to the reference genome (Additional file 1, Table S4, [GenBank: HM149237, HM149238, HM149239, HM149240, HM149241, HM149242, HM149243, HM149244, HM149245, HM149246, HM149247 and HM149248].). We identified a homozygous four bp deletion in affected dogs, relative to both Border collie controls and the dog reference genome. The sequence of the affected allele is a deletion from bases 4411956 to 4411960 on chromosome 13 (Dog Genome Build 2.1), which is listed as being within exon 19 of *VPS13B *(g.4411956_4411960delGTTT, [GenBank: HM036106]). Obligate carriers (known to have produced affected offspring) were heterozygous for the four bp deletion (Additional file 1, figure S3). The deletion results in frame shift of putative transcript variants 1, 3 and 4 and premature truncation after the 980^th ^amino acid of the 4019, 3994 and 1427 amino acid *VPS13B *protein isoforms, respectively (figure 1).

We sequenced exon 19 in 69 Border collies (six affected, 10 obligate carriers and 47 of unknown status), and all affecteds were homozygotes for the GTTT deletion and all obligate carriers were heterozygotes (Table 2). The deletion co-segregated with the TNS phenotype in all cases. Of the 47 Border collies related to the TNS pedigree with unknown status, 29 were heterozygous and 18 were homozygous for the normal sequence. We designed a PCR assay to detect the deletion based on the size of the PCR product.

**Table 2:** Genotyping of Border collies for the *VPS13B *deletion for dogs from the TNS pedigree, a set of Australian Border collies unrelated to TNS affected lineages, and a set of dogs collected randomly from Norway.

status	homozygote deletion	heterozygote	homozygote normal
affected	6	-	-

Obligate carrier	-	10	-

related†	-	29	18

unrelated‡	-	40	220

Norway*	1	9	70

† ancestors and siblings of TNS affected dogs

‡ Australian Border collie sample set collected prior to TNS work

* sample set collected randomly to represent Border collie population of Norway

We applied the TNS carrier test to a sample set of 260 Australian Border collies collected before the TNS research began. These samples can be considered randomly sampled with respect to TNS. We also tested a set of 80 samples that were collected in Norway to represent the local Border collie population (phenotype data was not collected, Table 2). The sample sets gave allele proportions of 0.07 and 0.08, respectively, for the TNS mutation. We have tested 5000 Border collies from Europe, USA, UK, Australia, New Zealand and Japan, 1100 were carriers and 30 were affected. This sample set of 5000 has a strong ascertainment bias towards dogs with familial links to known carriers. To correct for this ascertainment bias, we removed any samples where either of the parents had been tested as carriers, leaving 2100 dogs. Allele frequencies ranged from 0.04 to 0.08 per country with an average of 0.064 from the sample set of 2100 dogs (Additional file 1, Table S5).

A comparison between descriptions of Cohen syndrome patients in the literature and seven TNS affected Border collies identified an overlap in disease characteristics (Table 3). Features common to both Cohen syndrome and TNS include: typical facies; slender extremities; infantile hypotonia; developmental delay; and neutropenia. However, obesity and ophthalmic abnormalities, which are other common Cohen syndrome clinical signs, were not found in Border collies either because they are not present or could not be examined (several affecteds are deceased and were reported on posthumously). Mental retardation is a clinical sign in Cohen syndrome that is difficult to assess in dogs, but inability to learn simple commands was reported by the breeder for three of the seven affecteds dogs while some dogs were reported to be quick learners.

**Table 3:** Comparison of clinical signs with percentages between Cohen syndrome patients and seven TNS affected Border collies.

Clinical signs	**Cohen Syndrome (%)**†	TNS (%)
typical facies	81*	43

slender extremities	91	86

mental retardation	99	43

microcephaly	53	71

infantile hypotonia	86	71

developmental delay	81	86

delayed puberty	41	86

short stature	47	60‡

truncal obesity	65	0

neutropenia	75§	100

myopia	44	n/a

cataracts	64	n/a

n/a - not available

* average for clinical signs: short philtrum, high narrow palate, prominent nasal bridge and prominent central upper incisors

† Data summarised from Taban *et al. *[23] where the number of individuals for each category varies depending on what has been reported in the literature

‡ out of five dogs as two died before reaching maturity

§ data summarised from Hennies et al. [24]; Kivitie-Kallio and Norio [25]; Chandler et al. [26]; Kolehmainen et al. [15]; Bugiani et al. [27]; Kivitie-Kallio et al. [12]; and Seifert et al. [14].

To determine whether the mutation is causative rather than linked to a non-coding *VPS13B *mutation or mutation in another gene in the region we compared sequence data from other species. High conservation of *VPS13B *is observed at both the amino acid and DNA level across mammalian species, for example human - dog homologies 90.4% for amino acids, 90.7% for mRNA; human - mouse 87.3% for amino acids, 86.5% mRNA; dog - mouse 85.6% for amino acids, 84.8% mRNA. A multiple species comparison using PhastCons from the UCSC genome browser shows the most highly conserved regions to be exons and transcription factor binding sites, which suggests conservation in the regulation of *VPS13B*.

**Figure 2:**
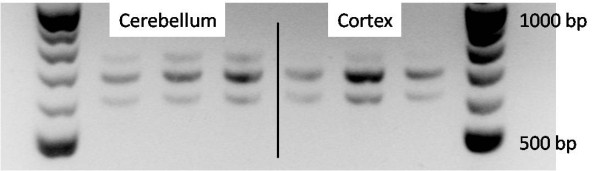
**PCR product from cerebellum and cortex of three healthy dogs for primers spanning exon 25-28 of cDNA**. Product 609 bp and 684 bp is present corresponding to mRNA incorporating exons 27b or 27, respectively. Lanes one and eight contain a 100 bp ladder, lanes two to four are cerebellum samples and five to seven are cortex samples from three healthy dogs.

There is a large *VPS13B *transcript variant in human that is not detected in mouse. The two largest putative transcripts of *VPS13B *in the dog are variant 1 (XM_539102) and variant 3 (XM_850840) which utilise an alternative exon 27, referred to as exon 27b. To investigate the presence of these transcript variants in dog brain tissue, we amplified cortex and cerebellum cDNA from three healthy dogs by PCR using primers spanning the alternate exon region (Additional file 1, Table S6). We found products of 684 bp corresponding to transcript variant 1 and 609 bp corresponding to transcript variant 3 to be present in both tissue types (Figure 2). The PCR fragment for transcript variant 1 showed higher intensity on the gel suggesting higher expression levels. A product of approximately 750 bp was also present on the gel at a lower concentration in cerebellum and was barely detectable in cortex. We sequenced the PCR product mixture and found sequence homozygosity up to the expected alternate exon site followed by mixed sequence. The mixed sequence could be resolved to the expected component sequences of the two transcripts confirming that the bands on the gel were the expected PCR products from the transcript variants. Sequence from the 750 bp product was not detectable in the sequence results from cortex or cerebellum, clear PCR termination sites were identified at 589 bp and 664 bp from the primer binding site.

We used comparative genomics to identify other species capable of this alternative splicing by aligning reference genome sequences from NCBI and canine exons 27 and 27b. We observed that human, cow, horse, mouse and rat all have an exon that is 67 bp in length and highly similar sequence to exon 27b in dogs (Additional file 1, Figure S4). Human, horse and cow, but not mouse or rat, have an exon 142 bp in length that is highly similar in sequence to exon 27 in dogs. For this exon, the mouse reference genome (C57BL/6J) had 80% identity with a seven bp deletion compared to dog. The rat (BN/SsNHsd) showed 80% identity for the first 59 bp of the sequence (Additional file 1, Figure S4), but a 271 bp deletion, relative to the mouse, disrupts the sequence (Additional file 1, Figure S5).

## Discussion

The high frequency of the TNS deletion in the Border collie population suggests that the mutation is much older than the recent common ancestor of our affected cases [7]. The mutation is present in Border collies in all countries tested and in both the working dog and show dog subgroups, which have been genetically isolated for around 50 years. The widespread nature of the disease indicates that the mutation was either already present in the founder dogs used to establish the breed or originated very early in the breed. If the mutation predates development of the breed it may exist in other collie breeds, as does Collie Eye Anomaly [13]. The sample sets collected from Norway and Australia prior to TNS research represent an unbiased sample set giving the most reliable disease allele proportion of 7-8%. The allele proportions from other countries after correction for ascertainment bias support the values obtained from these two sample sets.

We have shown that TNS in Border collies is caused by a mutation in the same gene that causes Cohen syndrome in humans, *VPS13B*. Over 80 mutations in human *VPS13B *have been identified, all resulting in the characteristic Cohen phenotype. The highly conserved nature of *VPS13B *suggests that the mutation identified in the canine form of the gene would cause similar clinical signs to that observed in human. The strong similarity in clinical signs between Cohen syndrome and TNS is supporting evidence that *VPS13B *is the TNS gene. We have found no reports of mutations in this gene in other organisms which makes TNS in the dog the first model for Cohen syndrome.

Cohen syndrome is rare (~190 patients worldwide) with a homogenous phenotype reported in Finnish patients, who carry mainly the c.3348_3349delCT mutation, and large variation in clinical signs in non-Finnish patients who carry many different mutations [11,14]. Diagnosis of Cohen syndrome is based on the presence of at least six of the following main clinical signs: developmental delay; microcephaly; typical Cohen syndrome facial gestalt; truncal obesity with slender extremities; overly sociable behaviour; joint hypermobility; high myopia and/or retinal dystrophy; and neutropenia [15]. Border collies show developmental delay; microcephaly; typical facial gestalt; slender extremities; and neutropenia, which was enough overlap for us to suspect TNS was homologous to Cohen syndrome.

The Cohen syndrome gene, *VPS13B*, is a potential transmembrane protein involved in vesicle-mediated transport and sorting within the cell [11]. The gene is similar to yeast *Vps13 *and is most highly conserved at the ends of the protein which are similar to vacuolar protein sorting domains [11]. *Vps13 *has undergone duplication events early in vertebrate evolution resulting in four paralogous copies *VPS13A*, *B*, *C *and *D*, which have diverged in function. The transcripts of each of the four genes typically vary by a few amino acids in the middle of the sequence and exhibit tissue specific expression. Each has one ubiquitous transcript and one transcript expressed in brain [16]. Human *VPS13B *has four transcript variants, two large transcripts utilising 62 exons each and two small transcripts utilising eight and 18 exons.

The canine form of *VPS13B *is located at approximately 4.1 Mb on canine chromosome 13 (CFA13) and contains 63 exons with five putative transcript variants (NCBI Dog Build V2.1). Seifert *et al*. [17] found that the full length transcript variants showed the highest expression with smaller transcripts barely detectable in both humans and mice. This finding suggests that the smaller transcripts are either from imperfect splicing or play a regulatory role in *VPS13B *expression. It is also likely that the smaller of the five putative transcripts listed for canine *VPS13B *have similar expression and functions. The two largest transcripts of *VPS13B *in humans utilise alternate splicing to include either exon 28 (NM_017890) or 28b (NM_152564) which are homologous to dog exons 27 and 27b, respectively. In humans, transcript NM_152564 is ubiquitous and transcript NM_017890 is expressed at roughly 20% of the concentration compared to kidney, placenta, small intestine, and lung, while in the brain and retina both transcripts have roughly equal expression [17].

Seifert *et al*. [17] have shown that mice lack a transcript homologous to human NM_017890, and only the ubiquitous transcript is detectable. We have shown that dogs express both transcripts in the brain incorporating exons 27 or 27b. We also detected a weak product at around 750 bp which may correspond to the incorporation of both exons 27 and 27b. The product was too weak to be detected in mixed sequence in either cerebellum or cortex. An mRNA incorporating both exons would change the reading frame and considering the low levels of expression is likely to be a splicing artefact.

We used comparative genomics to show that cow and horse are also likely to express both transcripts. The mouse and rat were found to have independent deletions disrupting use of the exon homologous to canine exon 27. In the case of the mouse, utilising this exon would change the reading frame and destroy protein function. The deletion in the rat removes the 3' half of the exon destroying the splice signal. This shows that the alternate transcript is not required in either mouse or rat.

Mental retardation observed in Cohen syndrome suggests that *VPS13B *plays a role in brain development or maintaining brain function in humans. Seifert *et al*. [17] hypothesized that disruption to the transcript utilising exon 28 may be responsible for the mental retardation observed in Cohen syndrome. The lack of mutations discovered in humans that involve sequence from either exon 28 or 28b make it difficult to test this hypothesis. Dogs express both transcript variants in the brain and an equivalent of mental retardation is observed in some TNS affected dogs. This identifies dogs as a good model to test the hypothesis that the human transcript utilising exon 28 may be responsible for mental retardation.

## Conclusion

We have shown that TNS in Border collies is the result of a mutation in *VPS13B *and that dogs, like humans, express alternately spliced transcripts of *VPS13B *in the brain. We have used comparative genomics to show that other mammalian species are likely to express alternately spliced transcripts in the brain, similar to human and dog. Dogs affected with TNS are the first animal model for Cohen syndrome and can be used to study the development of the disease and the effect at the cellular level of varying expression of *VPS13B*.

## Methods

We extracted DNA from Border collie blood samples following a standard salting out method [18]. The pedigree of affected dogs used in this study is published in Shearman and Wilton [7]. We designed primers for microsatellite amplification and *VPS13B *sequencing (Additional file 1, Tables S1 and S6) based on the canine genomic sequence (NCBI Dog Build V2.1) using Primer3 [[19], http://frodo.wi.mit.edu/primer3/.

We developed primers for four new microsatellites from the *VPS13B *region using the reference canine sequence (build 2.1) (Additional file 1, Table S1). We chose microsatellites with a minimum of 20 repeat units of two to four bp in the reference sequence and designed amplification primers. We named microsatellites based on chromosome number and location in units of 10 kb. C13.0390 (CFA13 at 390 × 10^4 ^bp) is approximately 197 kb before the transcription start site of *VPS13B*, C13.0423 is in intron 16, C13.0449 is in intron 24 and C13.0478 is in intron 43. Additional microsatellites were used from dbSTS (NCBI) (Additional file 1, Table S1). We used the universal priming method [20,21] to label PCR products which were sized on an ABI3730 DNA analyser (Applied Biosystems) with size standards GS500 and analysed using ABI Genemapper software (Applied Biosystems). We performed Linkage analysis using Superlink Online [22].

We designed primers to intron sequences to capture the entire exon with intron-exon boundaries (Additional file 1, Table S6). We performed sequencing using ABI BigDye chemistry V3.1 (Applied Biosystems) and an ABI3730 DNA analyser at the Ramaciotti Centre for Gene Function Analysis. We aligned all exon sequences to the reference dog genome and used Seqscape (Applied Biosystems) to compare carriers and affecteds to control Border collie sequences.

We designed primers flanking the deletion (Additional file 1, Table S6) to develop a carrier test based on sizing the PCR product on an ABI 3730 DNA Analyzer to identify presence of the deletion.

The clinical sign analysis was based on a yes/no questionnaire, with a short description required for yes answers, that was completed by owners of affected dogs. The questions were based on clinical signs observed in Cohen syndrome affected humans.

To detect presence of the alternately spliced transcripts in dog we designed primers in exons 25 and 28 (Additional file 1, Table S6) to produce a product incorporating alternately spliced products of exon 27 (XM_539102) or 27b (XM_850840). We expected the PCR product sizes from transcripts XM_539102 and XM_850840 using these primers to be 684 bp and 609 bp, respectively, based on the reference genome sequence. We prepared the cortex and cerebellum cDNA from three healthy dogs. We removed cortex tissue first and cerebellum tissue approximately 10 minutes later from dogs immediately after euthanasia and placed the samples into liquid nitrogen. We extracted RNA using the RNeasy Lipid Tissue Mini Kit from Qiagen and prepared cDNA using poly-T primers and PCR. We performed PCR on the cDNA, ran the PCR product on a 1% agarose gel and sequenced the PCR mix using the above sequencing method.

## Authors' contributions

JS participated in the design of the study, performed the experimental work, analysed the data and drafted the manuscript. AW conceived the study and participated in its design, coordination and data analysis and helped to draft the manuscript. All authors read and approved the final manuscript.

## Supplementary Material

Additional file 1 **Includes six supplementary figures and six supplementary tables**: Figure S1: Multipoint LOD scores for linkage to TNS gene by microsatellites C13.0390, C13.0423, C13.0449, C13.0478 in *VPS13B *region on CFA3. Figure S2: Genotypes at four microsatellite loci near *VPS13B *in eight TNS affected border collies identified by Dog ID number. Figure S3: Comparison of sequence electropherograms for an affected, carrier and control Border collie showing four bp deletion in exon 19 of *VPS13B *for bases 2894 to 2897 (transcript variant 1). Figure S4: Sequence comparison of *VPS13B *exons 27 and 27b in human to dog, horse, cow, rat and mouse showing high conservation for non-rodents. Figure S5: Sequence comparison between mouse and rat of *VPS13B *sequence homologous to exon 28 in human showing 271 bp deletion in rat. Table S1: Microsatellite primers used for linkage analysis of candidate genes. Table S2: Microsatellite allele sizes in 72 Border collies for the four microsatellites in the *VPS13B *region. Table S3: Non-disease haplotypes for the four microsatellites in the *VPS13B *region in 72 Border collies. Table S4: Single nucleotide variations identified from sequencing genomic DNA of *VPS13B *showing their genomic location, predicted effect on the protein produced for transcript variant 1, dbSNP ID where available and whether the SNP is in LD with TNS. Table S5: TNS Test results showing allele proportions by country after correcting for ascertainment bias. Table S6: Primers used to amplify exons from genomic DNA and from cDNA variants, and sequence PCR products Click here for file
