# Supplementary material for: A canine model of Cohen syndrome: Trapped Neutrophil Syndrome
Source: BMC Genomics. 2011 May 23;12:258. doi: 10.1186/1471-2164-12-258 (PMC3128065; doi:10.1186/1471-2164-12-258)
Supplement: Additional file 1 — Includes six supplementary figures and six supplementary tables: Figure S1: Multipoint LOD scores for linkage to TNS gene by microsatellites C13.0390, C13.0423, C13.0449, C13.0478 in VPS13B region on CFA3. Figure S2: Genotypes at four microsatellite loci near VPS13B in eight TNS affected border collies identified by Dog ID number. Figure S3: Comparison of sequence electropherograms for an affected, carrier and control Border collie showing four bp deletion in exon 19 of VPS13B for bases 2894 to 2897 (transcript variant 1). Figure S4: Sequence comparison of VPS13B exons 27 and 27b in human to dog, horse, cow, rat and mouse showing high conservation for non-rodents. Figure S5: Sequence comparison between mouse and rat of VPS13B sequence homologous to exon 28 in human showing 271 bp deletion in rat. Table S1: Microsatellite primers used for linkage analysis of candidate genes. Table S2: Microsatellite allele sizes in 72 Border collies for the four microsatellites in the VPS13B region. Table S3: Non-disease haplotypes for the four microsatellites in the VPS13B region in 72 Border collies. Table S4: Single nucleotide variations identified from sequencing genomic DNA of VPS13B showing their genomic location, predicted effect on the protein produced for transcript variant 1, dbSNP ID where available and whether the SNP is in LD with TNS. Table S5: TNS Test results showing allele proportions by country after correcting for ascertainment bias. Table S6: Primers used to amplify exons from genomic DNA and from cDNA variants, and sequence PCR products [file 1471-2164-12-258-S1.DOC]

**Supplementary Figures and Tables**

Figure S1 Multipoint LOD scores for linkage to TNS gene by microsatellites C13.0390, C13.0423, C13.0449, C13.0478 in *VPS13B* region on CFA3.

|  | **Dog ID** | | |  |  |  |  |  |  |  |  |  |  |  |  |  |
| --- | --- | --- | --- | --- | --- | --- | --- | --- | --- | --- | --- | --- | --- | --- | --- | --- |
| **Microsatellite** | **537** |  | **541** |  | **576** |  | **577** |  | **621** |  | **893** |  | **1137** |  | **1151** |  |
| **C13.0390** | 277 | 277 | 277 | 290 | 277 | 277 | 277 | 290 | 277 | 277 | 277 | 277 | 277 | 277 | 277 | 277 |
| **C13.0423** | 384 | 384 | 384 | 384 | 384 | 384 | 384 | 384 | 384 | 384 | 384 | 384 | 384 | 384 | 384 | 384 |
| **C13.0449** | 386 | 386 | 386 | 386 | 386 | 386 | 386 | 386 | 386 | 386 | 386 | 390 | 386 | 386 | 386 | 386 |
| **C13.0478** | 195 | 195 | 195 | 195 | 195 | 195 | 195 | 195 | 195 | 195 | 195 | 195 | 195 | 195 | 195 | 195 |

Figure S2: Genotypes at four microsatellite loci near *VPS13B* in eight TNS affected border collies identified by Dog ID number.


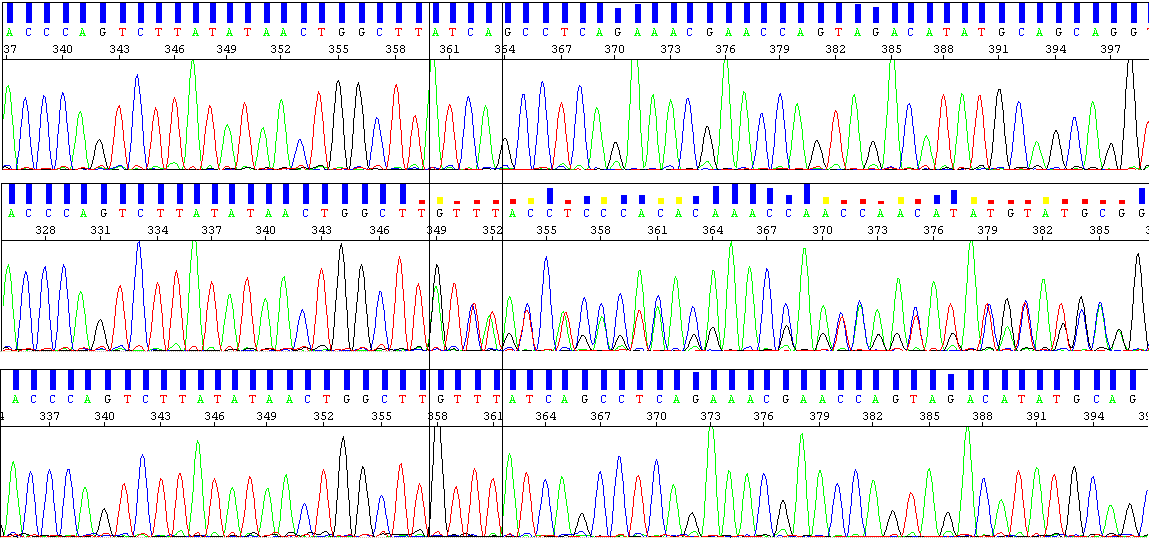


Affected

Control

Carrier

Figure S3: comparison of sequence electropherograms for an affected, carrier and control Border collie showing four bp deletion in exon 19 of *VPS13B* for bases 2894 to 2897 (transcript variant 1).

**exon 27**

>Dog 1 CTTTGGGGAAGAGTGTTGGTCTCTGGGGCAATGTGGAGGTGTCTTCCTTTCCTGTACTGACAAGCTGAACAGACGCACCT

>Human 1 CCTTGGGGAAGAGTGTTGGTCTTTGGGGCAATGTGGAGGTGTCTTCCTTTCCTGTACTGACAAGCTGAACAGACGCACCT

>Horse 1 CCTTGGGGAAGAGTGTTGGTCTCTGGGGCAATGTGGAGGTGTCTTCCTTTCCTGTACTGACAAGCTGAACAGACGCACCT

>Cow 1 CCTTGGGGAAGAGTGTTGGTCTCTGGGGCAATGTGGAGGTGTCTTCCTTTCCTGTACTGACAAGCTGAACAGACGCACCT

>Rat 1 TCTTGGGGAAGAGTGTTGGTCTTTGGGGGAGTGTGGAGGTGTCT------------------------------------

>Mouse 1 CCTTGGAGAAGAGTGTT-------GGGGCACCGTGGAGGTGCCTTCCTTTCCTGTACTGAGTAGCTGGACACTTGCACCT

>Human 81 TGTTGGTTCGACCCATCAGCAAGCAGGACCCTTTCAGTAATTGCTCTGGCTTCTTTCCTTCT

>Dog 81 TGTTGGTTCGACCCATCAGCAAGCAGGACCCTTTCAGTAATTGCTCTGGCTTCTTTCCTTCT

>Horse 81 TGTTGGTTCGACCCATCAGCAAGCAGGACCCTTTCAGTAATTGCTCTGGCTTCTTTCCTTCT

>Cow 81 TGTTGGTTCGACCCATCAGCAACCAGGACCCTTTCAGTAATTGCTCTGGCTTCTTTCCTTCT

>Rat 81 --------------------------------------------------------------

>Mouse 81 TGTTGGTTCGACCCCTTAGCAAGCAGGACTCTTGAAGTAACTGTCCTGCCTTCCTTTCTTCT

**exon 27b**

>Dog 1 GCCAGGGGAAGGTTGGCAGTCAGGACATTTTGAAGGAGTATTTCTACAGTGCAAAGAAAAACCTGTG

>Human 1 GCCAGGGGAAGGTTGGCAGTCAGGACATTTTGAAGGAGTATTTCTACAATGCAAAGAAAAATCTGTG

>Horse 1 GCCAGGGGAAGGTTGGCAGTCAGGACATTTTGAAGGAGTATTTCTACAGTGCAAGGAAAAACCTGTG

>Cow 1 GCCAGGGGAAGGTTGGCAGTCAGGACATTTTGAAGGAGTATTTCTACAGTGCAAAGAAAAACCTGTG

>Rat 1 GCCTGGGGAAGGTTGGCAATCAGGACAATTTGAAGGAGTGTTTCTGCAGTGCAAAGAAAAACCTGTG

>Mouse 1 GCCTGGGGAAGGTTGGCAGTCAGGACATTTTGAAGGAGTGTTTCTGCAGTGCAAAGAAAAACCTGTG

Figure S4: Sequence comparison of *VPS13B* exons 27 and 27b in human to dog, horse, cow, rat and mouse showing high conservation for non-rodents.

Mouse CCTTGGAGAAGAGTGTTGG-------GGCACCGTGGAGGTGCCTTCCTTTCCTGTACTGA

Rat TCTTGGGGAAGAGTGTTGGTCTTTGGGGGAGTGTGGAGGTGTCT----------------

Mouse GTAGCTGGACACTTGCACCTTGTTGGTTCGACCCCTTAGCAAGCAGGACTCTTGAAGTAA

Rat ------------------------------------------------------------

Mouse CTGTCCTGCCTTCCTTTCTTCTGTAAGAAATCACTTTAAAGCTGTACCCCAGAATTCCCC

Rat ------------------------------------------------------------

Mouse CTAGCAAGTACCTAAAATAGGACCTCAGCTGCCATACAGTCATCACTTGATTTTCATTGT

Rat ------------------------------------------------------------

Mouse TGCCAATGAAATACTCTTTGAAATAGAGTGAACTATAATCTGTTTTTGTATCATAAATTT

Rat ------------------------------------------------------------

Mouse GTTCTGTCATGTGCTGATTGCCA-TCTGTATGTA-TAT-AGCTAGT-CTGAGATTGTGAG

Rat ---------------GGTTGTCAGTCTGTATTTACTATCA-CT-GTTCTGAGTTTGTGAG

Mouse TAAGAGTTAATATTGTCATCAATATAAGAAAATCAAAGTAAGTTATTAAGTAAAATGGCT

Rat TAGGAATTAATATTGCCATCAGTAGAAGAAAGTTAAAGTAAGTTATTAAGTAAAATGGCT

Mouse TTCTACTTGTGTTGAAC

Rat TTTTACTTGTGTTGAAT

Figure S5: Sequence comparison between mouse and rat of *VPS13B* sequence homologous to exon 28 in human showing 271 bp deletion in rat.

Table S1: Microsatellite primers used for linkage analysis of candidate genes

| **Gene** | **Microsatellite** **name** | **Forward** **sequence** | **Reverse** **sequence** | **Repeat type** |
| --- | --- | --- | --- | --- |
| VPS13B | C13.0390 | TGGAGCTCTGAAGCTAGTAAGGA | TCCAAAGCTGGAAACAGAGAA | AAAGG x 21 |
|  | C13.0423 | GACACTGGCTTTTCTGTTGTTC | GGCTAATGAAACATTTGCTTTGT | TTTC x 39 |
|  | C13.0449 | ATCATTTTGGTGGCATCCAT | TGAAATATTGCTTTGTTCAAAACTT | TTTC x 28 - CT x 13 |
|  | C13.0478 | TGGGTGTTTTCAGGTTGAAG | TGCTTCTTGTCTGTCATGGAA | TA x 30 |
| BCL2L1 | FH3616* | GTGGGGAGTCTGTTTGTCC | CAACGGAGTCAGGAGTATAACC | AAAG x 19 |
|  | FH2495* | ATTTCATATGTGAGGCTGAGATTG | CAGTGGGAGAAAGATGCCAT | AAAG x 15 |
|  | REN127K17* | TATTTCTGCTATTTGTGTC | ATTCCATGTAGATTGTCA | TG x 11 |
| CXCL12 | FH2759* | AGTACTTGAGGCTTGGAGTCAG | CAAGCTGAGAGCCATGTAGG | GT x 23 |
|  | C28.176* | TCAGGCTCTCCAGAGACACA | TTGCCAATAGCAGACTGTGG | CA x 13 |
|  | REN205C12* | TGTTCTCATCCCATGCTTCC | TAGCTGCCTGATGGGGTAAC | GT x 12 |
| CXCR2 | REN41P15* | GTTCCGGTTATTGCGTGTG | CTGGGTCGGAGGAAGATG | AC x 10 |

* Microsatellites from dbSTS (NCBI)

Table S2: Microsatellite allele sizes in 72 control Border collies for the four microsatellites in the *VPS13B* region

| **Microsatellite** | **Allele sizes** | |  |  |  |  |  |  |  |  |  |  |  |  |  |  |  |  |
| --- | --- | --- | --- | --- | --- | --- | --- | --- | --- | --- | --- | --- | --- | --- | --- | --- | --- | --- |
| C13.0390 | 259 | 269 | 274 | 277 | 279 | 282 | 283 | 288 | 292 | 293 | 303 | 307 | 312 | 313 |  |  |  |  |
| C13.0423 | 336 | 338 | 342 | 345 | 352 | 356 | 369 | 374 | 377 | 382 | 389 | 393 | 398 | 410 | 415 | 418 | 444 | 457 |
| C13.0449 | 368 | 372 | 376 | 378 | 380 | 384 | 386 | 388 | 390 | 392 | 396 |  |  |  |  |  |  |  |
| C13.0478 | 183 | 185 | 187 | 189 | 193 | 195 | 199 | 205 |  |  |  |  |  |  |  |  |  |  |

Table S3: Non-disease haplotypes for the four microsatellites in the *VPS13B* region in 72 Border collies

|  | **Non disease haplotypes** | | |  |  |  |  |  |  |  |  |
| --- | --- | --- | --- | --- | --- | --- | --- | --- | --- | --- | --- |
| C13.0390 | 259 | 269 | 274 | 274 | 279 | 282 | 283 | 283 | 283 | 283 | 288 |
| C13.0423 | 410 | 374 | 418 | 415 | 352 | 345 | 356 | 352 | 356 | 356 | 444 |
| C13.0449 | 380 | 372 | 378 | 378 | 388 | 392 | 384 | 288 | 392 | 388 | 384 |
| C13.0478 | 185 | 193 | 185 | 185 | 183 | 195 | 183 | 183 | 183 | 183 | 189 |
|  |  |  |  |  |  |  |  |  |  |  |  |
| C13.0390 | 288 | 288 | 288 | 288 | 293 | 293 | 303 | 303 | 307 | 313 | 313 |
| C13.0423 | 457 | 342 | 357 | 349 | 398 | 382 | 374 | 377 | 393 | 369 | 369 |
| C13.0449 | 380 | 376 | 380 | 380 | 372 | 368 | 396 | 396 | 376 | 396 | 396 |
| C13.0478 | 187 | 187 | 187 | 187 | 183 | 199 | 193 | 193 | 205 | 195 | 193 |

Table S4: Single nucleotide variations identified from sequencing genomic DNA of *VPS13B* showing their genomic location, predicted effect on the protein produced for transcript variant 1, dbSNP ID where available and whether the SNP is in LD with TNS

| **Exon†** | **Variant identified** | **aa Effect^‡^** | **GenBank #** | **dbSNP ID** | **Comment*** |
| --- | --- | --- | --- | --- | --- |
| 6 | g.4159271T>A | syn | HM149237 | rs22238678 | Not in LD with TNS |
| 8 | g.4176521T>C | syn | HM149238 | rs22238688 | Not in LD with TNS |
| 11 | g.4178874G>A | G549S | HM149239 | rs22238710 | Not in LD with TNS |
| 16 | g.4232838A>T | Q813H | HM149240 | n/a | Not in LD with TNS |
| 19 (18) | g.4319973G>A | syn | HM149241 | n/a | Not in LD with TNS |
| 37 (35) | g.4676737G>A | C2096Y | HM149242 | n/a | Not in LD with TNS |
| 43 (41) | g.4744210A>G | syn | HM149243 | rs22277807 | Not in LD with TNS |
| 45 (43) | g.4772965G>A | R2712Q | HM149244 | n/a | Not in LD with TNS |
| 52 (50) | g.4789089C>T | syn | HM149245 | rs22310936 | Not in LD with TNS |
| 57 (55) | g.4812943G>A | A3434T | HM149246 | n/a | Not in LD with TNS |
|  | g.4813323C>T | syn |  | rs22253538 | Reference allele in LD with TNS |
| 59 (57) | g.4819760C>A | syn | HM149247 | n/a | Not in LD with TNS |
| 63 (61) | g.4833061A>G | syn | HM149248 | n/a | Variant allele in LD with TNS |

† Genomic exons numbered 1-63. Numbers in brackets represent exon numbering for transcript variant 1

‡ aa effect based on predicted mRNA sequence of transcript variant 1 (Genbank: XM_539102.2), syn = synonymous

* only dogs with known relatedness to TNS cases were tested, it is possible that the alleles identified as being in LD with TNS also occur in haplotypes that lack the TNS deletion in the larger population

Table S5: TNS Test results showing allele proportions by country after correcting for ascertainment bias

| **Country** | **Not TNS** | **TNS carrier** | **TNS affected** | **Total** | **TNS allele proportion** | **Carrier Rate** |
| --- | --- | --- | --- | --- | --- | --- |
| Australia^1^ | 220 | 40 | 0 | 260 | 0.077 | 0.154 |
| Norway^2^ | 71 | 9 | 1 | 81 | 0.068 | 0.111 |
| Japan^3^ | 69 | 14 | 0 | 83 | 0.084 | 0.169 |
| Finland^3^ | 120 | 18 | 0 | 138 | 0.065 | 0.130 |
| Czeck Rep^3^ | 55 | 8 | 0 | 63 | 0.063 | 0.127 |
| Netherlands^3^ | 136 | 12 | 0 | 148 | 0.041 | 0.081 |
| Germany^3^ | 223 | 33 | 0 | 256 | 0.064 | 0.129 |
| UK^3^ | 665 | 85 | 0 | 750 | 0.057 | 0.113 |
| US^3^ | 263 | 52 | 0 | 315 | 0.083 | 0.165 |
| Total | 1822 | 271 | 1 | 2094 | 0.065 | 0.129 |

^1^ Samples collected before TNS research

^2^ Random sample of unrelated dogs

^3^ Samples tested for TNS excluding any with either parent tested as TNS carriers

Table S6: Primers used to amplify exons from genomic DNA and from cDNA variants, and sequence PCR products

| **sequencing** **primers**  ***VPS13B*** | **Forwards primer** | **Reverse primer** |
| --- | --- | --- |
| Exon 1 | AGTTGCAGCTGGAGTAGACACAG | AACAAACCATCACTAGTAACGAAGC |
| Exon 2 | TGTGTGAACCTTTGTTATGTACCAC | AATAGGCATGTGAGAAACAGTAAGTG |
| Exon 3 | TTTTAAACGCTGTTTGAGAATGC | GTAAGTTAAAATCCTGCTGAAGAGC |
| Exon 4 | CCAGTTTTTGTAAGCCTGTCTACC | TACTACACTTGAAACAGGCATGTGG |
| Exon 5 | ATAAATGAGAGATTCGTTGCATAGG | ACATCTGGACAAACTTAAAAGGAAG |
| Exon 6 | AGTGAACTGTTTTGGCTTATATTGG | AAAAATCAAAGTAGTGTCAAACAACC |
| Exon 7 | CCAACAAACTTGAAAGTAGTAATGC | AACAAACTTCAAATCAGATCCTTCC |
| Exon 8-9 | TGGTATTTCAGTTCTCTCTTTGAGC | TCCCTGTATTCTATCCATAAAGAGG |
| Exon 10 | TTTTCTTTAACAGTTTAATGATTTTTCC | TCTTCAAACTTCTCTAGATGTAGAACC |
| Exon 11 | TAGAATTACGGATGTCCTTTGG | AGAACCAACTGATTCTGATGAGG |
| Exon 12 | GAGAATTGGTTTGAAGGCATTAAG | AACTAGCAAGAGACTCAAAATTAGGC |
| Exon 13 | AAAGTATAATTTGACCTTGAAATTTGG | CACAGTTACATTGCTTTAACACACC |
| Exon 14 | TTCTAGATTGATTTGATGACATTGC | TTACATATCATTTAAATCTCATCTTATGG |
| Exon 15 | TAGTAGGAAGCAATGATTTTTCAGG | ATACCCACCAACACTTAACACTACC |
| Exon 16 | TCTTTCTGAAGATTGTCTAGATTGAAAG | TTACCTCGCATATCCTGAATTTTAC |
| Exon 17 | GGTGATTCTAACAAAAACTTAATAAGAGG | ATGTCACTACCCTCTTTCCTAGTCG |
| Exon 18 | ATTAAGTTTAAATGCCTTGGTGAAG | CAGTAACGCTCCTTGTAAAATGC |
| Exon 19 | CTTACTTTGTTAAGAATCAAAAGTGC | AGTTTCATAGAGCACAACTGTAGGG |
| Exon 20 | TATGAATACTGCCTTGAAAAGTTGG | TTAAACTAGTCTCTCCAGTGTTTCG |
| Exon 21 | CCAAGGGAGCTACATATTGATTG | TCATGTCTCTTTAGCTGGCATTC |
| Exon 22 | CTGACAAGAGTAACCAACACATTCC | ATAAAGTGCACCAAGAATTTTCACC |
| Exon 23 | GTTACTCATAAAGGCATGAATATGG | ACATCAATATGATTAAAAGGTGTGG |
| Exon 24 | GCTGCATTAAAAATGGTTTTACCTC | CTTATCTAACCACGCTGATCTTGAC |
| Exon 25 | TTAATTGTGATTCGAAGTTGAAAGG | AGAAACATTAATAAACAGCCACTGC |
| Exon 26-27 | TCGTTAGCATTTATGATATTGAAAAG | GACTGTGTTCAAAGAAAGTATAGCTC |
| Exon 28-29 | TCAGTGTAGTGTTGTTTACATAATAACC | TCATTGACAAAGTAGATGATACAGAGG |
| Exon 30 | TCAAGGGATGTAGACATAATAAAAGG | ACACCAAAGTGCAAACTACAACC |
| Exon 31 | TTTAATTCATGTGTTTTAATGTTGG | CTTCTTTACAGGATGAAATTGTTGC |
| Exon 32 | TCCAATGTGGTTACTTCCTAATACC | AAAAGTGGTACCCAAACACTTAGG |
| Exon 33 | CAAATATAACTGTCATACATGTTTGTGG | TAATAAGCAATTTGACATTCTGTGC |
| Exon 34 | ATTAAACTATGTTGAGTGGTTGTGC | TAATAGAGGCTTTTCTGAGGGTAGG |
| Exon 35 | ACCATTTTAACCTCTTTATTACATCC | TTGATCGATCTTGATACAATGG |
| Exon 36 | TGAGTACATACAAGAACATGCAAGG | ACTATTTATTTTTGGCAAGGAATGC |
| Exon 37 | GCTTACATTCAATGACAGAGCTTC | TGGATACAAACACTTTAGTTTACCG |
| Exon 38 | CAACTGAAAAAGACTCTGACAAAGG | TCAATCTGATAATACTCTTTCTCACC |
| Exon 39-40 | TGCATTAATTCTAGTGTGATTGTGG | TTTCCTAGTATATAATCAGCACTTTTCC |
| Exon 41 | AATATGCATTTCAACAAATACAGAAC | ATCTGTAGATCACACGGATGCAG |
| Exon 42 | CATAAGTTGTGTGATATGTGGATGG | TCACAGCACTTTGAAACATCAATAC |
| Exon 43 | GTAATGCCTTCAAGAGAGTGTTAGG | AGAGTGCCTAGTAAAGATCACATGC |
| Exon 44 | AAACATCTTGCAATTAATCTGATGC | GAATAAATGAATCCCCAACCTTAAC |
| Exon 45 | TGACATATTTGCTATTCTTAGCTTGG | GCAAGGTAGTAGTAAGCGTACATAGG |
| Exon 46 | CAGGCACACAACCAGTATTTCC | AAACTACTGGGACTGATACAACAGC |
| Exon 47-48 | TCTATCAACCTCTCCTCACACAAAC | AACGGTACAAGGTTGCTTTTCTAC |
| Exon 49 | GTCCCTAAATCAACAAAAATTAGCC | ACACCTCTTCACACTAGAATCATCC |
| Exon 50 | ATTGGATGATTCTAGTGTGAAGAGG | GAATATCAAAGAACATTCCATACCC |
| Exon 51 | TGTACCTAAGGTTGCATATTATTCTTTAC | TTGTCTAGAGCATGTAATACTAAAAAGG |
| Exon 52 | GAACACAAGTTAAAATTGGTACTATCC | GATTTCTGCTCATTAACTACCTTGG |
| Exon 53 | AGGATACGTGGTTATGGTTTCTTAG | CACTGCAGCTACTCTTTTGTCTCTC |
| Exon 54-55 | CAAATCTAAATAAACAGGTACCAAGC | TATAAAAATGGATTCACAGGTGTCC |
| Exon 56 | ATAGAACTAGCTGTTGAGCCAGAAC | GATGCTAATATGTAATTCGAAGATGC |
| Exon 57 | AGGGTGAGACTGATGTTCTTCC | AGGTCTTGAGGGCGATAAGG |
| Exon 58 | GTCAGAGTAAAGCTATTTGCTAGGC | GGAATAACAGTTGAGCCAATCC |
| Exon 59 | ATTGTCTTAAGGCCCTTGCTTC | GCTCACTGTGGAGCACGAAC |
| Exon 60 | TGATGAAGATCATAAGTGTCTTAGTAACC | TAAGAATGCAAGTTAGTCAGTGAGG |
| Exon 61 | TGTAAACAGATGACATCACTACAGC | AAGGTCACCTCTGTTAGAATGTGG |
| Exon 62 | TGGTAAAGTGAGTGCAAATACACG | ATGACAGGCTCCAATACACACC |
| Exon 63 | TACTCAGATGTATCAAGGCAAAAGC | TGTGTTAGAGCAGCGAGTTAACAG |
| **Exon 27 Splice variant** |  |  |
| Exon 25-28 | GATCCAGTGGACGTATTAGTCTGTG | GTAAACTGCGTGAAGTCAATGTATG |
